# Hydrodissection of the Eighth Cervical Nerve Root for Scapular Girdle Pain Associated With Nonunion of the First Rib Stress Fracture: A Case Report

**DOI:** 10.7759/cureus.60156

**Published:** 2024-05-12

**Authors:** Toru Omodani

**Affiliations:** 1 Orthopaedics, Tokyo Advanced Orthopaedics, Tokyo, JPN

**Keywords:** scapular girdle pain, cervical nerve root, ultrasound, hydrodissection, first rib stress fracture

## Abstract

The first rib stress fracture is a rare overuse injury, with nonunion posing challenges to athletic performance. We report an 18-year-old international-level gymnast diagnosed with the nonunion of the first rib stress fracture, experiencing pain extending to the medial scapular area. Traditional treatments provided no relief, with tests suggesting C8 nerve root involvement. This study introduces a novel approach targeting the C8 nerve root using hydrodissection, which alleviates the pain. Post-procedure, the patient resumed competition without recurrence of pain after a year. This case suggests that pain due to first rib stress fracture nonunion might be associated with the C8 nerve root, and hydrodissection could be a potentially effective treatment.

## Introduction

A stress fracture of the first rib is one of the overuse injuries that occur in the trunk and is a rare pathology [1]. Patients typically report pain in the scapular region [2]. First-rib stress fractures are often seen in baseball players, and it is believed that the accumulation of stress on the first rib from batting and pitching is one of the causes of this condition. Conservative treatment is generally considered to have good outcomes. However, it can take some time for bone union and recovery. In some cases, complications such as nonunion can arise, complicating the return to sports activity [3]. If a stress fracture does not heal and symptoms persist, partial resection of the first rib may be performed.

To date, there have been no reports of injections administered for pain associated with nonunion of the first rib stress fracture. We report a case where hydrodissection of the eighth cervical (C8) nerve root was effective for pain associated with non-union of the first rib stress fracture.

## Case presentation

**Figure 1:**
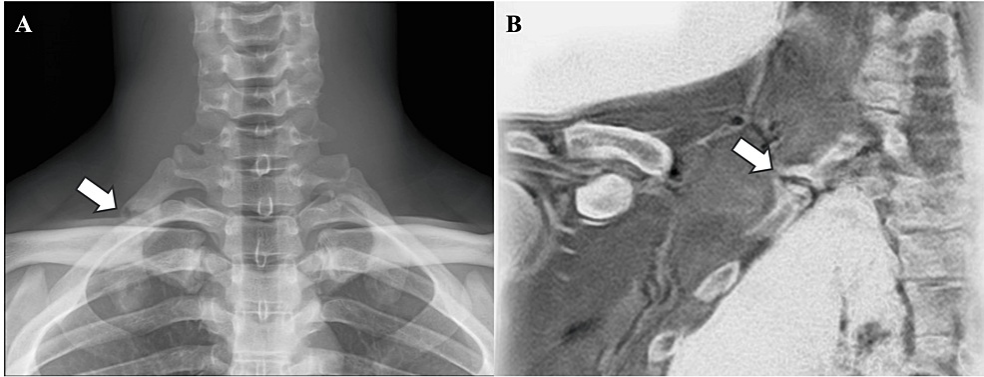
Image findings of the first rib stress fracture The nonunion of the first rib stress fracture was confirmed by radiograph (A) and bone magnetic resonance imaging (B). The arrow indicates a discontinuity in the first rib.

An 18-year-old male, an international-level gymnast, began to experience pain in his right shoulder extending to the medial scapular area during competitions. There were no complaints of muscle weakness in the upper limb. A month after the onset of symptoms, he visited a local hospital where, based on plain radiographs and bone magnetic resonance imaging (MRI), he was diagnosed with a right first rib stress fracture. Despite adjusting his training volume and continuing with rehabilitation, there was no improvement in pain during competitions, leading to a decline in his performance. A year after the onset of his symptoms, he visited our institution. The radiographs and MRI confirmed that the first rib stress fracture had developed into nonunion (Figure 1). Movement of the shoulder joint did not induce any pain. Jackson's test and Spurling's test, suggesting symptoms of cervical nerve root involvement, were positive, and these tests elicited pain from the right shoulder to the medial scapula. Due to the suspicion of C8 nerve root symptoms, it was decided to proceed with hydrodissection of the C8 nerve root.

**Figure 2:**
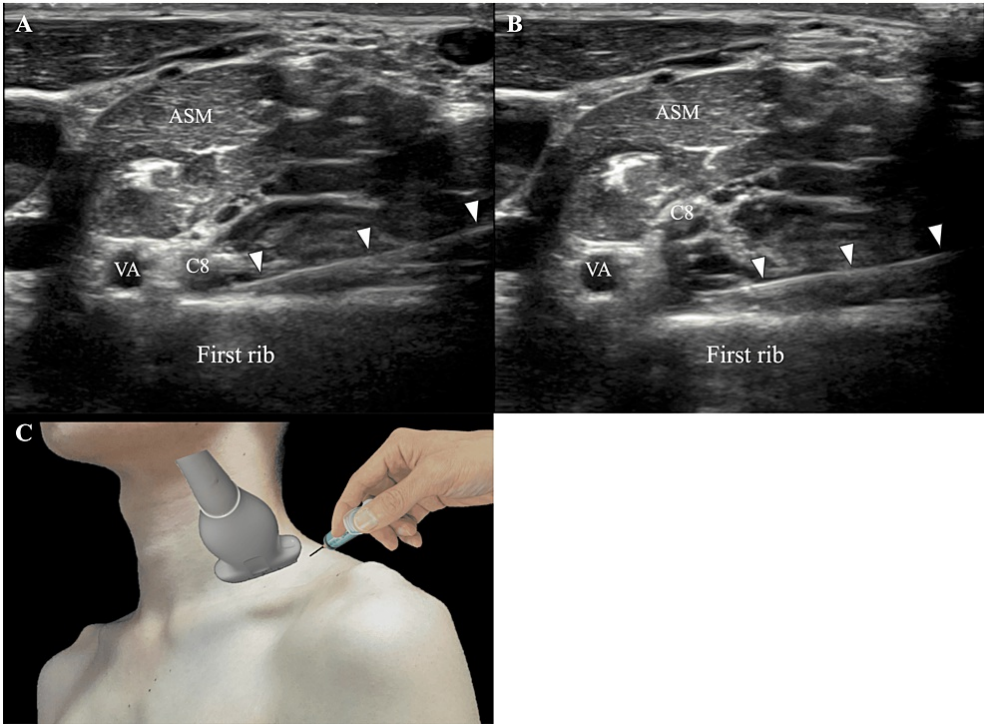
Hydrodissection of the eighth cervical (C8) nerve root A: A 25-gauge, 60-mm needle was inserted and advanced between the C8 nerve root and the first rib; B: Hydrodissection was performed between the C8 nerve root and the first rib; C: The probe was placed just proximal to the clavicle, and the needle was inserted from the posterior neck Arrowheads: needle, VA: vertebral artery, C8: eighth cervical nerve root, ASM: anterior scalene muscle

The patient was positioned in the lateral decubitus position on the bed with the right side up. The ultrasound probe was placed on the right cervical region in a short-axis orientation. The C8 nerve root, which exits from the intervertebral foramen of the eighth cervical vertebra and runs over the first rib, was identified by ultrasound. From the posterior neck, a 25-gauge, 60-mm needle was inserted and advanced between the C8 nerve root and the first rib. Using 5 ml of 0.09% lidocaine diluted in saline, hydrodissection was performed between the C8 nerve root and the first rib (Figure 2, Video 1). An ultrasound machine from Canon Medical Systems (Tochigi, Japan), the Aplio i700, was used with an 18-MHz probe. Immediately post-injection, both the Jackson's test and the Spurling's test were negative. No complications arose from the injection, and the patient resumed his practice the following day.

**Video 1:** Hydrodissection of the eighth cervical nerve root

After the injection, the pain during the competition disappeared. One year post-injection, the patient continues to compete without any recurrence of pain.

## Discussion

In this case study, the focus for pain management was not on the first rib itself but rather on the C8 nerve root. The C8 nerve root exits the spinal canal from the intervertebral foramen of the eighth cervical vertebra and then passes between the first rib and the clavicle as it proceeds distally [4]. Pathologies that can impede the eighth cervical nerve root include cervical intervertebral disc herniation, cervical rib, pseudarthrosis following fractures of the clavicle, and fractures of the first rib [5]. Im et al. reported a case presenting with C8 nerve root symptoms associated with a fracture of the first rib, where Sparling's test was positive. Similar physical findings were observed in this case as well, and they were consistent with previous reports [6]. In this case, the bone overgrowth associated with the pseudarthrosis of the first rib stress fracture, combined with soft tissue proliferation, might have caused some form of compression or adhesion to the C8 nerve root, potentially resulting in radicular symptoms.

Hydrodissection is a technique that involves fluid separation between tissues using a medicinal solution [7]. When specifically targeting nerves, this method is referred to as nerve hydrodissection [8]. Hydrodissection is believed to be effective in alleviating pain of neural origin by reducing nerve glide resistance and improving local circulation around the nerve [9]. In this case, the pain was alleviated by performing a hydrodissection of the C8 nerve root. It is believed that the primary pathology was not the pain from the nonunion of the first rib itself but pain originating from the nearby running C8 nerve root. The improvement in pain through hydrodissection further supports this hypothesis. Lin et al. have reported on the efficacy and safety of ultrasound-guided hydrodissection for cervical nerve roots. There were cases where the patients experienced dizziness after the injection, but the symptoms disappeared within four hours in all cases. No serious complications such as nerve damage or vascular injuries occurred. In this case as well, it is believed that precise injections under ultrasound guidance may have prevented serious complications [10].

The novelty of this case lies in targeting the C8 nerve root for pain management, rather than focusing on the first rib itself. The potential effectiveness of hydrodissection of the C8 nerve root as a treatment for pain associated with nonunion of the first rib stress fracture was suggested.

## Conclusions

This study introduces a novel approach targeting the C8 nerve root using hydrodissection, which alleviates the pain. Post-procedure, the patient resumed competition without recurrence of pain after a year. This case suggests that pain due to first rib stress fracture nonunion might be associated with the C8 nerve root, and hydrodissection could be a potentially effective treatment. This is a case report, and further research is needed in the future.

## References

[1] Alderson BR (1947). Further observations on fracture of the first rib. Br J Radiol.

[2] Mithöfer K, Giza E (2004). Pseudarthrosis of the first rib in the overhead athlete. Br J Sports Med.

[3] Funakoshi T, Furushima K, Kusano H (2019). First-rib stress fracture in overhead throwing athletes. J Bone Joint Surg Am.

[4] Zhu YS, Mu NN, Zheng MJ (2014). High-resolution ultrasonography for the diagnosis of brachial plexus root lesions. Ultrasound Med Biol.

[5] Ferrante MA, Ferrante ND (2017). The thoracic outlet syndromes: part 2. The arterial, venous, neurovascular, and disputed thoracic outlet syndromes. Muscle Nerve.

[6] Im YJ, Kang MS, Kim SW, Sung DH (2021). Brachial plexus injury associated with median sternotomy during cardiac surgery: three cases of C8 radiculopathy due to the fracture of the first rib. Diagnostics (Basel).

[7] Cass SP (2016). Ultrasound-guided nerve hydrodissection: what is it? A review of the literature. Curr Sports Med Rep.

[8] Andreone BJ, Lacoste B, Gu C (2015). Neuronal and vascular interactions. Annu Rev Neurosci.

[9] Lam KH, Hung CY, Chiang YP, Onishi K, Su DC, Clark TB, Reeves KD (2020). Ultrasound-guided nerve hydrodissection for pain management: Rationale, methods, current literature, and theoretical mechanisms. J Pain Res.

[10] Lin CH, Yen YS, Wu CY (2023). Ultrasound-guided nerve hydrodissection of cervical nerve roots for cervical radicular pain in patients with mild and moderate to severe stenosis: a retrospective cohort study. Sci Rep.

